# Estimation of a Within-Herd Transmission Rate for African Swine Fever in Vietnam

**DOI:** 10.3390/ani13040571

**Published:** 2023-02-06

**Authors:** Van Phan Le, Nguyen Thi Lan, Jose Tobias Canevari, Juan Pablo Villanueva-Cabezas, Pawin Padungtod, Thi Bich Ngoc Trinh, Van Tam Nguyen, Caitlin N. Pfeiffer, Madalene V. Oberin, Simon M. Firestone, Mark A. Stevenson

**Affiliations:** 1 Faculty of Veterinary Medicine, Vietnam National University of Agriculture, Hanoi 10000, Vietnam; 2 Faculty of Veterinary and Agricultural Sciences, The University of Melbourne, Parkville 3010, Australia; 3 Department of Infectious Diseases, Peter Doherty Institute for Infection and Immunity, University of Melbourne, Parkville 3000, Australia; 4 One Health Unit, The Nossal Institute for Global Health, The University of Melbourne, Parkville 3010, Australia; 5 Food and Agriculture Organization of the United Nations, Hanoi 10000, Vietnam; 6 Institute of Veterinary Science and Technology, Hanoi 10000, Vietnam

**Keywords:** infectious diseases, epidemic, outbreak, virus, transboundary, mathematical modeling, simulation, epidemiology, livestock, panzootic

## Abstract

**Simple Summary:**

We describe results from a panel study in which pigs from a 17-sow African swine fever (ASF) positive herd in Thái Bình province, Vietnam, were followed over time to record the date of onset of ASF signs and the date of death from ASF. Our objectives were to (1) fit a susceptible-exposed-infectious-removed disease model to the data with transmission coefficients estimated using approximate Bayesian computation; (2) provide commentary on how a model of this type might be used to provide decision support for disease control authorities. Detailed datasets of epidemics afflicting smallholder herds are rare. This study provides insight into how ASF progresses in a small-scale semi-intensive pig herd in Vietnam and generated a dataset that facilitated the use of a modeling approach to describe the transmission dynamics in this frequently underreported sector.

**Abstract:**

We describe results from a panel study in which pigs from a 17-sow African swine fever (ASF) positive herd in Thái Bình province, Vietnam, were followed over time to record the date of onset of ASF signs and the date of death from ASF. Our objectives were to (1) fit a susceptible-exposed-infectious-removed disease model to the data with transmission coefficients estimated using approximate Bayesian computation; (2) provide commentary on how a model of this type might be used to provide decision support for disease control authorities. For the outbreak in this herd, the median of the average latent period was 10 days (95% HPD (highest posterior density interval): 2 to 19 days), and the median of the average duration of infectiousness was 3 days (95% HPD: 2 to 4 days). The estimated median for the transmission coefficient was 3.3 (95% HPD: 0.4 to 8.9) infectious contacts per ASF-infectious pig per day. The estimated median for the basic reproductive number, R_0_, was 10 (95% HPD: 1.1 to 30). Our estimates of the basic reproductive number R_0_ were greater than estimates of R_0_ for ASF reported previously. The results presented in this study may be used to estimate the number of pigs expected to be showing clinical signs at a given number of days following an estimated incursion date. This will allow sample size calculations, with or without adjustment to account for less than perfect sensitivity of clinical examination, to be used to determine the appropriate number of pigs to examine to detect at least one with the disease. A second use of the results of this study would be to inform the equation-based within-herd spread components of stochastic agent-based and hybrid simulation models of ASF.

## 1. Introduction

In late January 2019, a 20-sow family-owned piggery in Thái Bình province in Vietnam experienced acute onset of illness in pigs of all age classes, with morbidity and mortality rates reaching greater than 50% by early February. African swine fever (ASF) was confirmed in this herd on 19 February 2019 [1]. Up to 31 December 2019, all 63 provinces throughout Vietnam had been confirmed with ASF, with 8,537 of Vietnam’s 11,129 communes (77%) affected.

Blood, tissues, secretions, and excretions of sick and dead animals are the major source of the virus within ASF-positive herds [2]. Pigs that recover from either the acute or chronic form of ASF can become carriers [3], and indirect transmission of disease can occur through contact with ASF-contaminated feed and other fomites. Where incident ASF cases in a herd are detected early, and effective disease control measures are applied rapidly, the within-herd spread of disease can be quite low, with disease confined to only some sheds or pens on a farm [4].

Infectious disease models have been used to reproduce the observed incidence and prevalence of diseases of both humans and animals, including influenza [5], human immunodeficiency virus [6], smallpox [7], malaria [8], foot-and-mouth disease [9,10,11] and Ebola virus disease [12]. Disease models are important tools for developing an understanding of the behavior of the disease in complex environments and for assessing the impact of interventions to limit disease spread. For example, models incorporating disease control measures (e.g., vaccination, movement restrictions, depopulation) can be used to identify situations where specific interventions or combinations of interventions are likely to have the greatest effect [7,9,13,14]. The use of state-of-the-art modeling to incorporate limited data from resource-poor settings affected by livestock diseases remains a challenge [15].

Susceptible-exposed-infectious-recovered or susceptible-exposed-infectious-removed (both abbreviated SEIR) models [16,17] describe the number of susceptible, exposed (latent), and infectious individuals in a population and how these numbers change over time. In an SEIR model, transmission coefficients quantify the rate at which individuals transit from the susceptible to exposed, exposed to infectious, and infectious to recovered or removed states. Model transmission coefficients are of key importance in fit-for-purpose model development because they are a major determinant of the accuracy of model predictions [18]. Approaches for numerical estimation of disease model transmission coefficients include experimental studies [19] and observational studies in which individuals from a population are assessed for the presence of disease at regular intervals [20]. In this study, we describe results from an observational study in which pigs from a 17-sow ASF-positive herd in Thái Bình province in Vietnam were followed over time to record the date of onset of ASF signs and the date of death from ASF. Our objectives were to: (1) fit an SEIR (susceptible-exposed-infectious-removed) model to the data with transmission coefficients estimated using approximate Bayesian computation [21,22]; (2) provide commentary on how a model of this type might be used to provide decision support for disease control authorities.

## 2. Materials and Methods

### 2.1. Study Population and Data Collection

This was a panel study of 17 pigs that comprised a small, family-owned farm in Thái Bình province, approximately 100 kilometers to the east of Hanoi, Vietnam. The farm was typical of smallholder swine enterprises in this area of Vietnam, with animals of a given age and production class (gilts, weaners, growers, and finishers) sharing the same pen. Dry and lactating sows were kept in individual stalls with little or no opportunity to make direct physical contact with each other. The herd was comprised of five pregnant sows, three sows that were nursing piglets, and nine gilts. Following the onset of clinical signs characteristic of ASF on 15 March 2019 (day 0)—including high fever, anorexia, lethargy, cutaneous hemorrhages, vomiting, respiratory distress, coughing, abortion, and unwillingness to stand—the first and second authors visited the farm every 2 to 3 days to (1) examine animals for the presence of clinical signs (including recording of body temperature using a digital thermometer), (2) take blood samples for real-time polymerase chain reaction assay (RT-PCR) using the VDx ASFV Gene Diagnosis Kit (http://www.mediandiagnostics.com/, accessed on 15 November 2022) [1], and (3) record the date and details of death for pigs that died. 

### 2.2. Model Structure

**Figure 1:**
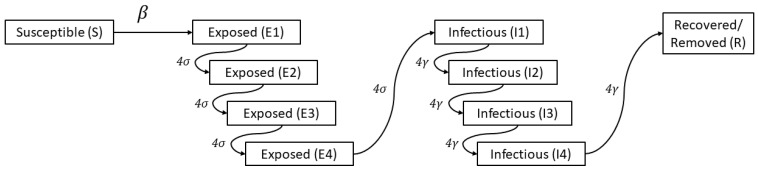
Schematic diagram of the susceptible-exposed-infectious-removed (SEIR) model described in the text. Sojourn times in the exposed I and infectious (I) states were split into 4 sub-compartments, effectively following Erlang distributions with shape parameter *k* = 4.

A frequency-dependent continuous time stochastic susceptible-exposed-infectious-removed model was developed (Figure 1) using the Gillespie algorithm [23,24]. The model was designed to estimate changes in the prevalence of infectious pigs as a function of the number of days following the onset of clinical signs of the assigned index case in the herd. Expressions defining the number of pigs transitioning out of the susceptible state into the exposed, infectious, and removed states as a function of time are shown in Equations 1 to 4, respectively.

(1) $$\frac{dS}{dt}=-\beta SI/N$$

(2) $$\frac{dE}{dt}=\beta SI/N-\sigma E$$

(3) $$\frac{dI}{dt}=\sigma E-\gamma I$$

(4) $$\frac{dR}{dt}=\gamma I$$

The rate at which pigs transitioned from the susceptible to the exposed state was dependent on a transmission coefficient, β. The rate at which pigs transitioned from the exposed to infectious state was dependent on σ such that the average latent period was equal to 1/σ. The rate at which individuals transition to the removed state was dependent on γ, defined as the inverse of the average duration of infectiousness [16]. The length of time individuals remained in the exposed (E), and infectious (I) states were assumed to follow Erlang distributions with a shape parameter k=4 which provides a good approximation to the latent and infectious length distributions [25]. With this approach, we allowed for a steeper increase in incidence and a shorter duration of the modeled outbreak [26], which captures the disease dynamics observed (Figure 1). 

### 2.3. Model Parameters

Approximate Bayesian computation (ABC) [27] methods using a Sequential Monte Carlo (SMC) algorithm to optimize computational efficiency [28,29] were used to estimate values of β, σ, and γ (Table 1) for the process that generated the available dataset. The bounds of the uniform prior distributions used in the ABC-SMC were set at values that encompassed the range of plausible values. These were based on a review of published literature from studies that reported on epidemiological parameters from field observations of African swine fever outbreaks (see Table 1), where possible, confined to estimates based only on within-farm transmission. The viral strains circulating in Vietnam at the time of the outbreak belonged to genotype II (p72 and p54 genes), serogroup 8 (CD2v gene), and CVR I – which share 100% identity with previously reported Vietnamese ASF viral isolates and those reported in China, Georgia, Korea, and Russia [30]. In order to perform an exhaustive exploration of the parameter space, the minimum and maximum limits of our prior distributions included parameter estimates derived from observational studies across different regions. The ABC SMC algorithm involved sampling combinations of β, σ, and γ values (i.e., particles) until n = 2000 particles were retained at each step, based on a distance function. The distance function was estimated as the difference between the observed data and three summary statistics for a simulation run on each particle: the number of days to reach the outbreak peak, the peak outbreak size, and the sum of epidemic curve residuals (i.e., the sum of the magnitude of the differences between the simulated and observed epidemic curve, by day). In the first step, particles were accepted if their distance from the observed data was within predefined tolerances, set to capture the peak within ± 4 days and ±4 cases, and an epidemic curve residual sum of ≤40 cases. The tolerance was progressively narrowed in sequential steps of the SMC algorithm to the 75th percentile of the distance from the observed data of the retained particles from the previous step, per summary statistic. The ABC-SMC algorithm was coded in R [31] and run until there was limited further gain with additional steps, in this case, for 18 steps. An estimate of the basic reproductive number ($R_{0}$), defined as the expected number of new infectious individuals that one infectious individual will produce during its period of infectiousness in a fully susceptible population [16], was obtained as a calculated output by dividing β by γ for each particle. All inferred parameters were reported based on the highest posterior density interval, using the contributed HDInterval package [32] in R. Research protocols for this study were approved by the Committee on Animal Research and Ethics, Faculty of Veterinary Medicine, Vietnam University of Agriculture (Approval No. CARE-201/04) and followed the principles regarding the privacy of personal data outlined in the Universal Declaration of Human Rights [33]. The data supporting the findings of this study were obtained with the oral consent of the farm owners and are available on request from the corresponding author. The data are not publicly available due to privacy reasons. 

## 3. Results

**Figure 2:**
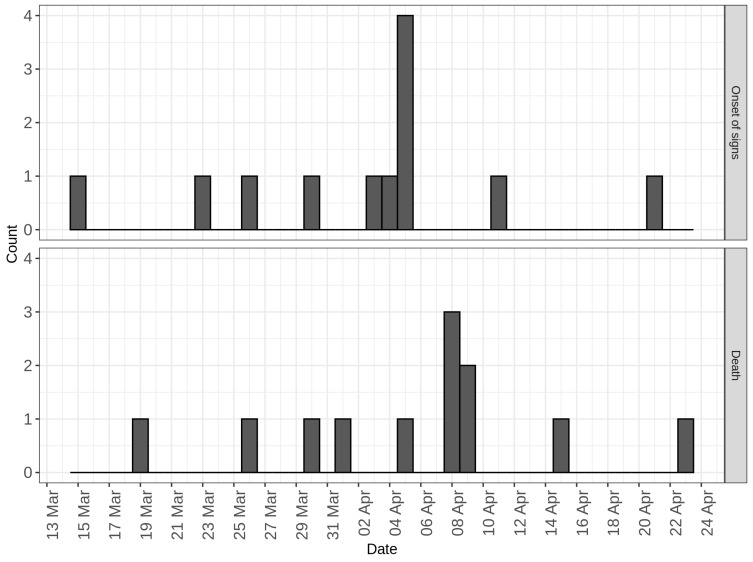
Frequency histogram showing counts of animals showing clinical signs and counts of animals that died as a function of calendar date.

A frequency histogram showing the date of onset of clinical signs and date of death as a function of calendar date is shown in Figure 2. The index case developed clinical signs on 15 March 2019 (day 0) and died four days later on 19 March 2019 (day 4). In total, 10 of the 17 adult pigs in the herd died from ASF. One pig with ASF was culled due to the severity of clinical signs. The last ASF death occurred on 23 April 2019 (day 39). The peak number of pigs with clinical signs occurred on 5 April 2019 (day 21, Figure 2). 

**Figure 3:**
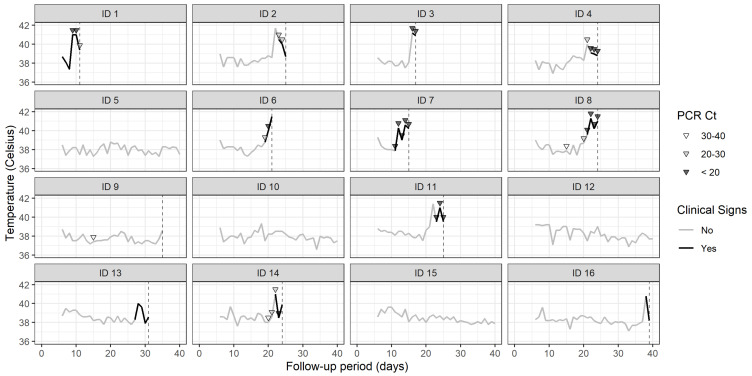
Line plots showing rectal temperature for individual pigs as a function of the number of days 15 March 2019. On each plot, the days on which pigs were showing clinical signs of ASF are shown in black. The days on which blood samples were taken from individual pigs are marked on each plot as inverted triangles, with shading indicating the PCR Ct value.

Figure 3 shows rectal temperatures for 16 of the 17 adult pigs in the herd from day 5 (20 March 2019) until the date of death or day 40 (24 April 2019), whichever came first. Eleven of the 16 pigs that were monitored developed fever, defined as a rectal temperature greater than or equal to 40 °C [2], and, of this group, nine died. One pig was culled, and one pig was alive on 24 April 2019 (day 40). A total of 22 real-time PCRs were carried out on 10 pigs, with positive results (PCR Ct < 35) returned on 22 occasions. One pig returned a positive test to PCR on 25 March 2019 (day 10) but did not develop a fever. This pig died on day 26 March 2019 (day 11).

**Figure 4:**
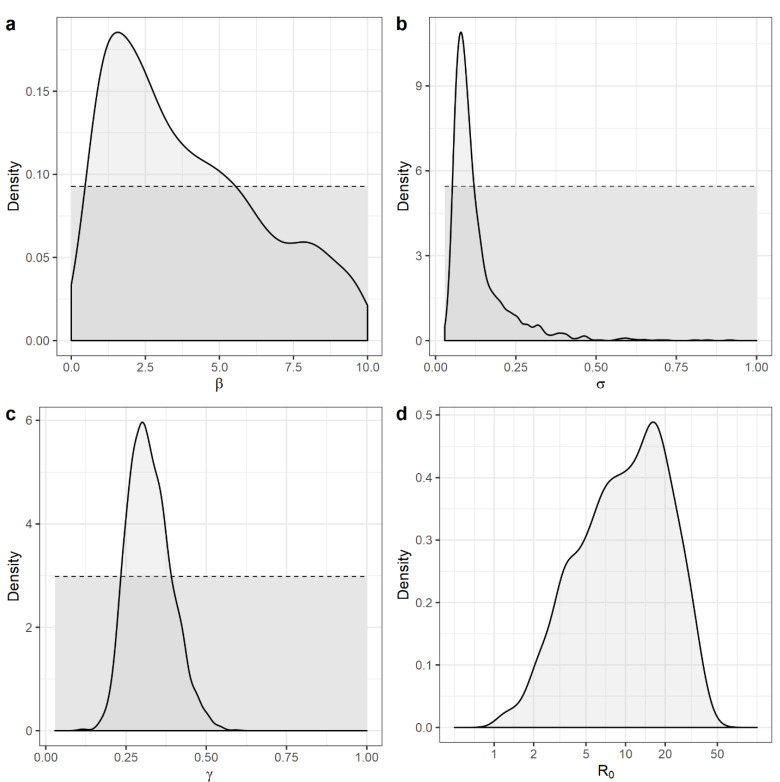
Prior (dashed lines) and posterior (solid lines) distributions for (**a**) the transmission coefficient β; (**b**) the inverse of the average latent period *σ*; (**c**) the inverse of the average duration of infectiousness *γ*; (**d**) the basic reproductive number, *R*_0_ which was a calculated output and (being a ratio) is presented on a logged horizontal axis.

**Figure 5:**
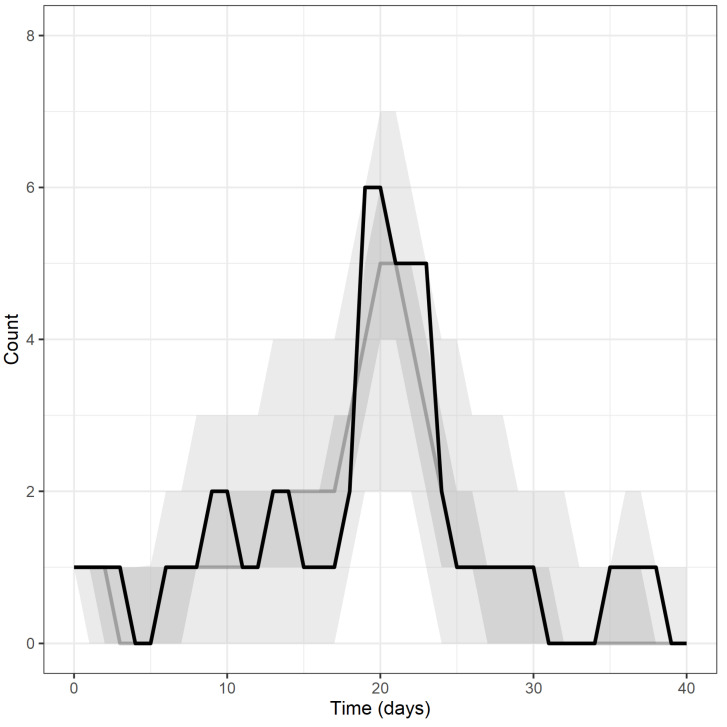
Line plot showing the number of infectious pigs as a function of the number of days since the onset of signs in the first affected pig (black line). Superimposed on this plot are the predicted median (grey line) number of infectious pigs per day. The dark and light shaded areas show the 50% and 95% quantiles around these estimates.

Descriptive statistics of the approximate posterior distributions of the transmission coefficient β, the average latent period $\sigma^{-1}$, the average duration of infectiousness $\gamma^{-1}$ and the basic reproductive number $R_{0}$ are presented in Table 2. Figure 4 shows the uniform prior distributions assigned to β, σ, and γ and the approximate posterior distributions estimated by the ABC-SMC algorithm, along with the calculated approximate posterior distribution for $R_{0}$, and Figure 5 shows the fit of the model predictive epidemic curve distributions to the empirical target distribution.

The approximate posterior distribution differed markedly from the prior distributions for all variables indicating that they were well informed by the data. For this outbreak, the median of the average latent period was 10 days (95% HPD [highest posterior density interval]: 2 to 19 days), and the median of the average duration of infectiousness was 3 days (95% HPD: 2 to 4 days). The estimated median for the transmission coefficient was 3.3 (95% HPD: 0.4 to 8.9) infectious contacts per ASF-infectious pig per day. The estimated median for $R_{0}$ was 10 (95% HPD: 1.1 to 30).

**Figure A1:**
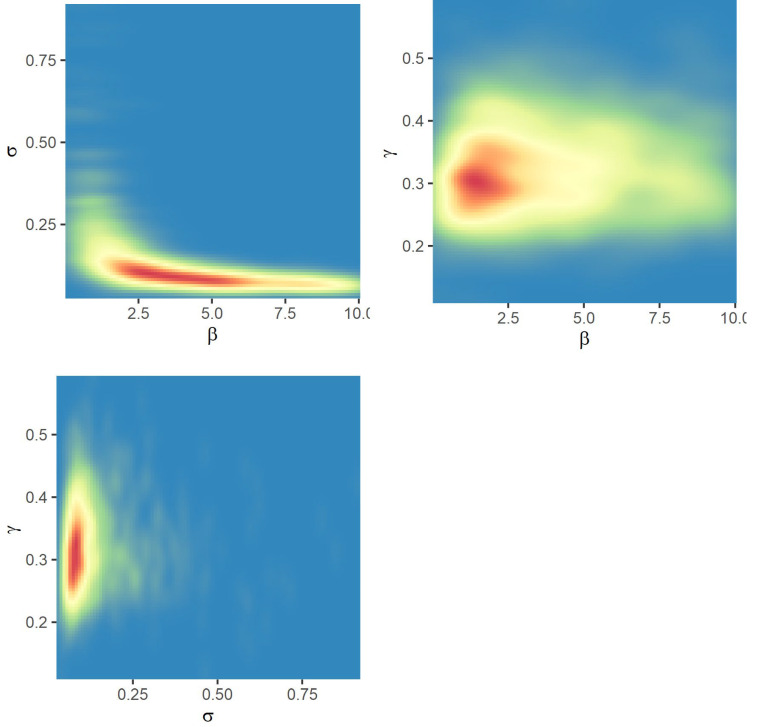
Heat map of the pair-wise correlations between parameters in the approximate posterior distribution.

**Figure A2:**
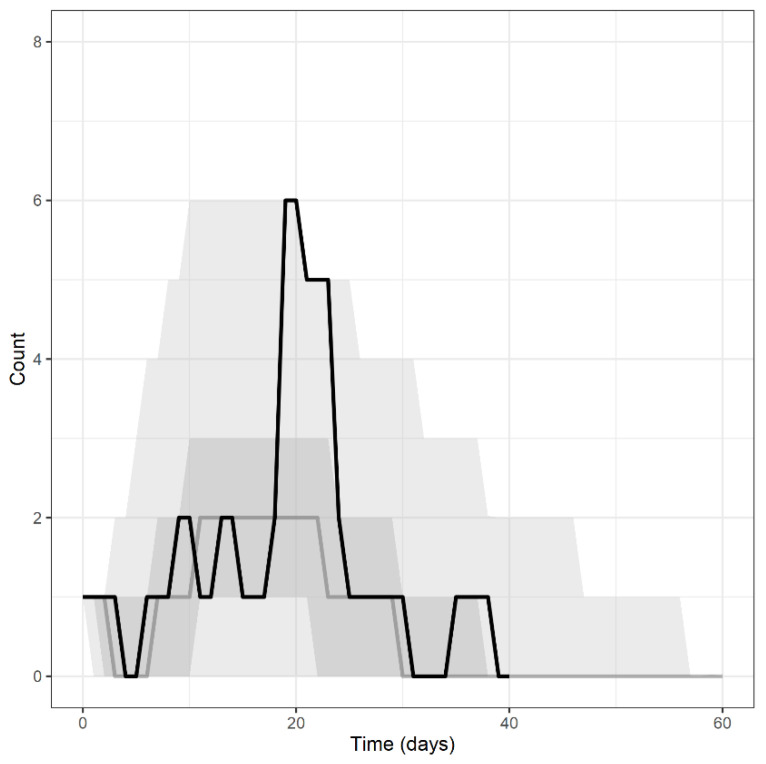
Line plots show the number of infectious pigs as a function of the number of days since the onset of signs in the first affected pig (black line). Superimposed on this plot are the predicted median (grey line) number of infectious pigs per day based on re-simulated samples from the approximate posterior. The dark and light shaded areas show the 50% and 95% quantiles around these estimates.

The Appendix A, Appendix B and Appendix C provides details of the full approximate posterior distribution, the R code used for these analyses, estimates of the cross-correlations between parameters in the accepted particles (Figure A1), and a comparison of trajectories of repeated simulations from the posterior distribution with the empirical data (Figure A2).

## 4. Discussion

ASF was officially announced in Vietnam in February 2019, leading to rapid detection, stamping out of ASF-infected farms, and strict monitoring of surrounding uninfected herds. Given the rapid spread of the virus and severe economic losses to farmers, the Department of Animal Health in Vietnam changed the policy and allowed spot elimination (also called “*pulling the tooth”*), which is the rapid detection and removal of ASF-infected animals while the rest of the herd remains under monitoring [39]. In this context, this investigation provides unique insight into how ASF progresses in a small-scale semi-intensive pig herd in Vietnam and generated data amenable to modeling approaches to describe the transmission dynamics in a type of premise frequently underreported in the literature [15]. 

Our modeling estimates provide useful starting points for modeling the spread of ASF within small-scale, semi-intensive Vietnamese pig herds. The estimated value of $R_{0}$ (10; 95% HPD: 1.1 to 30) in this study is comparable to the 5 to 12 range reported by Schulz et al. [40] in a study that summarized within-herd $R_{0}$ estimates across two observational studies [35,36]. Our median estimate of $R_{0}$ is also within the 4.92 and 24.2 range estimated under experimental conditions [41]. Estimates from field observations from an outbreak in 1977 in Ukraine [35] presented a lower range, from 6 to 9, while an analysis of a recent outbreak in Uganda, where the farming system is likely to differ markedly from Vietnam, was from 1.6 to 3.2 [38]. Differences in estimates of $R_{0}$ across studies arise from the properties of different virus isolates (e.g., virulence and infectivity), the frequency of contacts between pigs within a herd (influenced by herd structure and management), and the analytical method used [40]. A strength of this study is that empirical data were used to simultaneously estimate β, σ, and γ using ABC methods as opposed to the more commonly used and potentially biased technique that involves estimation of β using a generalized linear model and then assuming a fixed value for the duration of infectiousness, usually obtained from the literature.

Our estimate of *R_0_* is high and comparable to that of measles in human populations [42]. Thus, the pursuit of solutions based on vaccine development must also consider well-planned vaccine deployment to achieve high levels of coverage. In the absence of a vaccine, our results suggest that herd managers should regard all animals as potentially infectious in an ASF outbreak—not just those showing clinical signs. The time series plots of the rectal temperatures as a function of outbreak day, in combination with the results of PCR testing (Figure 3), show that pigs became PCR-positive and shed the virus before the onset of pyrexia and presumably clinical signs. These results agree with the findings of de Carvalho Ferreira et al. [41] in a study investigating the transmission rate of ASF under laboratory conditions. The application of strict biosecurity measures focused on minimizing the transference of infected body tissues and fomites from infected to uninfected animals [4] should be prioritized to reduce the within- and between-herd spread of ASF. Enforcement of strict biosecurity measures to mitigate ASF in smallholder premises requires close monitoring, as it may result in increasing trading and consumption of infected animals [43], magnifying the outbreak effects [44].

Thái Bình province is one of the most densely pig-populated areas in Vietnam per square kilometer, where the average number of pigs per farm is low (8 or less) [39]. The farm in our study could be considered typical of this province. A limitation of this investigation is that the parameters were estimated based on the disease dynamics observed in a single farm and do not capture the variability in breeds, herd size, infrastructure, management, and biosecurity across the Vietnamese swine sector. Moreover, the small population size resulted in large uncertainty (i.e., wide 95% HPD) in the parameters reported. 

We envisage two potential uses of the results presented. The first is at the individual farm level, in the situation when a herd manager may be concerned that ASF was introduced via (for example) shared, contaminated equipment. In this situation, a herd advisor could use our estimates of β, σ, and γ and the number of pigs at risk to determine the number of pigs expected to be showing clinical signs at a given number of days following the estimated incursion date. Sample size calculations, with or without adjustment to account for less than perfect sensitivity of clinical examination, could then be used to determine the appropriate number of pigs to examine to be 95% (or 99% confident) that at least one animal will be detected with disease [45]. A modification of this approach would be to calculate the expected duration of an outbreak if a herd in question was infected. If no deaths were encountered for the duration of the expected outbreak period, one could be confident that the herd was, in fact, ASF-free. A second use of the results of this study would be a modification of the equation-based within-herd spread components of stochastic agent-based and hybrid simulation models, such as the Australian Animal Disease (AADIS) model [46], to allow it to simulate the spread of ASF among domestic pigs, in addition to foot-and-mouth disease for which it was originally designed. 

## 5. Conclusions

The result of this study provides evidence that outbreaks of ASF in smallholder farms can be severe, leading to high mortality in a short period of time. In the absence of an effective vaccine or treatment for ASF, herd managers should contain their animals, monitor for clinical signs, report suspected outbreaks to authorities promptly, and undertake appropriate management of farm waste and deceased animal carcasses. The transmission parameters estimated provide quantitative insight into the epidemiology of ASF in the smallholder sector, which is frequently afflicted by ASF outbreaks but remains poorly understood. 

## Figures and Tables

**Table 1:** Description of model parameters and prior distributions used in the ABC-SMC, based on published information from empirical observations of within-farm spread of African swine fever.

Parameter	Description	Prior Use in ABC-SMC	Prior Published Ranges	Outbreak Location and Year, Strain Virulence, and References
*β*	Transmission coefficient	Uniform (0.001, 10)	0.7 to 2.2 0.8 to 1.3	Russia 2010–2014, moderate virulence [34] Ukraine 1977, high virulence [35]
*σ*	Inverse of the average latent period	Uniform (1/35, 1)	1/(5.8 to 9.7)	Russia 2010–2014, moderate virulence [34]
*γ*	Inverse of the average duration of infectiousness	Uniform (1/35, 1)	1/(4.5 to 8.3) 1/15 1/(13 to 19)	Russia 2010–2014, moderate virulence [34] Russia 2007–2010, moderate virulence [36] Nigeria 1997–2005, moderate virulence [37]
*R*_0_	Basic reproductive ratio	No prior calculated output	4.4 to 17 3.9 to 16 1.6 to 3.2 5.7 to 9.2	Russia 2010–2014, moderate virulence [34] Russia 2007–2010, moderate virulence [36] Uganda 2010–2011, high virulence [38] Ukraine 1977, high virulence [35]

**Table 2:** Descriptive statistics of the posterior distributions of the transmission coefficient β, the average latent period (days) $\sigma^{-1}$, the average duration of infectiousness (days) $\gamma^{-1}$ and the basic reproductive number $R_{0}$.

Parameter	Median (95% HPD)	1/Median (95% HPD)
β	3.3 (0.4, 8.9)	-
$\sigma^{-1}$	10.2 (2.1, 18.8)	0.10 (0.04, 0.31)
$\gamma^{-1}$	3.2 (2.0, 4.4)	0.32 (0.20, 0.45)
$R_{0}$	10.4 (1.1, 30.4)	-

## Data Availability

The data that support the findings of this study are available on request from the corresponding author. The data are not publicly available due to privacy reasons.

## Appendix A. Approximate Posterior Distribution and R Code

The full approximate posterior distribution and R code used for these analyses are provided here: https://doi.org/10.26188/12646541.

## Appendix C. Repeated Simulations from the Posterior Distribution Versus Empirical Data

The set of trajectories presented in Figure 5 in the main paper represents an asymptotic estimator of the posterior. A feature of ABC-SMC for such stochastic models is that repeated simulation of retained parameter values from the approximate posterior distribution can generate simulated trajectories that fall outside the range specified by the tolerance. The following figure presents 2000 re-simulated trajectories of particles sampled from the posterior in comparison with the empirical data. Of these trajectories, 19.6% met the tolerance criterion at the 20th step of the implemented ABC-SMC algorithm for the peak of the epidemic curve being captured within ±1 case, 13.3% met the tolerance criterion of the peak being captured ± 2 days, while 1.1% met the tolerance criterion of an epidemic curve residual sum of ≤29 cases. Although the observed realization fell within the 95% credible interval, the median simulated curve is much flatter, peaking earlier and lower. We consider this because the observed curve had an unusual shape for an SEIR model, with a long latent phase, then a very sudden peak and decline.

## References

[1] Le V.P., Jeong D.G., Yoon S.W., Kwon H.M., Trinh T.B.N., Nguyen T.L., Bui T.T.N., Oh J., Kim J.B., Cheong K.M. (2019). Outbreak of African Swine Fever, Vietnam, 2019. Emerg. Infect. Dis..

[2] Constable P., Hinchcliff K., Done S., Gruenberg W. (2016). Veterinary Medicine: A Textbook of the Diseases of Cattle, Horses, Sheep, Pigs and Goats.

[3] Sánchez-Vizcaíno J.M., Mur L., Martínez-López B. (2012). African Swine Fever: An Epidemiological Update. Transbound. Emerg. Dis..

[4] Depner K., Gortazar C., Guberti V., Masiulis M., More S., Olßsevskis E., Thulke H.H., Viltrop A., Wozniakowski G., Abrahantes J.C. (2017). Epidemiological Analyses of African Swine Fever in the Baltic States and Poland. EFSA J..

[5] Rvachev L.A., Longini I.M. (1985). A Mathematical Model for the Global Spread of Influenza. Math. Biosci..

[6] Anderson R.M., NG T.W., Boily M.C., May R.M. (1989). The Influence of Different Sexual-Contact Patterns between Age Classes on the Predicted Demographic Impact of AIDS in Developing Countries. Ann. N. Y. Acad. Sci..

[7] Ferguson N.M., Keeling M.J., Edmunds W.J., Gani R., Grenfell B.T., Anderson R.M., Leach S. (2003). Planning for Smallpox Outbreaks. Nature.

[8] Ngwa G.A., Shu W.S. (2000). A Mathematical Model for Endemic Malaria with Variable Human and Mosquito Populations. Math. Comput. Model..

[9] Morris R.S., Wilesmith J.W., Stern M.W., Sanson R.L., Stevenson M.A. (2001). Predictive Spatial Modelling of Alternative Control Strategies for the Foot-and-Mouth Disease Epidemic in Great Britain, 2001. Vet. Rec..

[10] Keeling M.J., Woolhouse M.E.J., Shaw D.J., Matthews L., Chase-Topping M., Haydon D.T., Cornell S.J., Kappey J., Wilesmith J., Grenfell B.T. (2001). Dynamics of the 2001 UK Foot and Mouth Epidemic: Stochastic Dispersal in a Heterogeneous Landscape. Science (1979).

[11] Ferguson N.M., Donnelly C.A., Anderson R.M. (2001). Transmission Intensity and Impact of Control Policies on the Foot and Mouth Epidemic in Great Britain. Nature.

[12] Wong Z.S.Y., Bui C.M., Chughtai A.A., MacIntyre C.R. (2017). A Systematic Review of Early Modelling Studies of Ebola Virus Disease in West Africa. Epidemiol. Infect..

[13] van Kerkhove M.D., Ferguson N.M. (2012). Epidemic and Intervention Modelling—A Scientific Rationale for Policy Decisions? Lessons from the 2009 Influenza Pandemic. Bull. World Health Organ..

[14] Williams A.D.C., Hall I.M., Rubin G.J., Amlôt R., Leach S. (2011). An Individual-Based Simulation of Pneumonic Plague Transmission Following an Outbreak and the Significance of Intervention Compliance. Epidemics.

[15] Brooks-Pollock E., de Jong M.C.M., Keeling M.J., Klinkenberg D., Wood J.L.N. (2015). Eight Challenges in Modelling Infectious Livestock Diseases. Epidemics.

[16] Keeling M.J., Rohani P. (2011). Modeling Infectious Diseases in Humans and Animals. Model. Infect. Dis. Hum. Anim..

[17] Begon M., Bennett M., Bowers R.G., French N.P., Hazel S.M., Turner J. (2002). A Clarification of Transmission Terms in Host-Microparasite Models: Numbers, Densities and Areas. Epidemiol. Infect..

[18] Kirkeby C., Halasa T., Gussmann M., Toft N., Græsbøll K. (2017). Methods for Estimating Disease Transmission Rates: Evaluating the Precision of Poisson Regression and Two Novel Methods. Sci. Rep..

[19] Bouma A., Claassen I., Natih K., Klinkenberg D., Donnelly C.A., Koch G., van Boven M. (2009). Estimation of Transmission Parameters of H5N1 Avian Influenza Virus in Chickens. PLoS Pathog..

[20] Backer J.A., Berto A., McCreary C., Martelli F., van der Poel W.H.M. (2012). Transmission Dynamics of Hepatitis E Virus in Pigs: Estimation from Field Data and Effect of Vaccination. Epidemics.

[21] Sunnåker M., Busetto A.G., Numminen E., Corander J., Foll M., Dessimoz C. (2013). Approximate Bayesian Computation. PLoS Comput. Biol..

[22] Beaumont M.A., Zhang W., Balding D.J. (2002). Approximate Bayesian Computation in Population Genetics. Genetics.

[23] Kendall D.G. (1950). An Artificial Realization of a Simple “Birth-And-Death” Process. J. R. Stat. Soc. Ser. B.

[24] Gillespie D.T. (1976). A General Method for Numerically Simulating the Stochastic Time Evolution of Coupled Chemical Reactions. J. Comput. Phys..

[25] Lloyd A.L. (2001). Realistic Distributions of Infectious Periods in Epidemic Models: Changing Patterns of Persistence and Dynamics. Theor. Popul. Biol..

[26] Wearing H.J., Rohani P., Keeling M.J. (2005). Appropriate Models for the Management of Infectious Diseases. PLoS Med..

[27] Tavaré S., Balding D.J., Griffiths R.C., Donnelly P. (1997). Inferring Coalescence Times from DNA Sequence Data. Genetics.

[28] Sisson S.A., Fan Y., Tanaka M.M. (2007). Sequential Monte Carlo without Likelihoods. Proc. Natl. Acad. Sci. USA.

[29] Liu J.S., Chen R. (2012). Sequential Monte Carlo Methods for Dynamic Systems. J. Am. Stat. Assoc..

[30] Nguyen V.T., Cho K.H., Mai N.T.A., Park J.Y., Trinh T.B.N., Jang M.K., Nguyen T.T.H., Vu X.D., Nguyen T.L., Nguyen V.D. (2022). Multiple Variants of African Swine Fever Virus Circulating in Vietnam. Arch. Virol..

[31] R: The R Project for Statistical Computing. https://www.r-project.org/.

[32] Meredith M., Kruschke J. Package “HDInterval” Type Package Title Highest (Posterior) Density Intervals 2022. https://cran.r-project.org/web/packages/HDInterval/index.html.

[33] (1948). United Nations Universal Declaration of Human Rights.

[34] Guinat C., Porphyre T., Gogin A., Dixon L., Pfeiffer D.U., Gubbins S. (2018). Inferring Within-Herd Transmission Parameters for African Swine Fever Virus Using Mortality Data from Outbreaks in the Russian Federation. Transbound. Emerg. Dis..

[35] Korennoy F.I., Gulenkin V.M., Gogin A.E., Vergne T., Karaulov A.K. (2017). Estimating the Basic Reproductive Number for African Swine Fever Using the Ukrainian Historical Epidemic of 1977. Transbound. Emerg. Dis..

[36] Gulenkin V.M., Korennoy F.I., Karaulov A.K., Dudnikov S.A. (2011). Cartographical Analysis of African Swine Fever Outbreaks in the Territory of the Russian Federation and Computer Modeling of the Basic Reproduction Ratio. Prev. Vet. Med..

[37] Olugasa B., Ijagbone I. (2007). Pattern of Spread of African Swine Fever in South-Western Nigeria, 1997–2005. Vet. Ital..

[38] Barongo M.B., Ståhl K., Bett B., Bishop R.P., Fèvre E.M., Aliro T., Okoth E., Masembe C., Knobel D., Ssematimba A. (2015). Estimating the Basic Reproductive Number (R0) for African Swine Fever Virus (ASFV) Transmission between Pig Herds in Uganda. PLoS ONE.

[39] Nga B.T.T., Padungtod P., Depner K., Chuong V.D., Duy D.T., Anh N.D., Dietze K. (2022). Implications of Partial Culling on African Swine Fever Control Effectiveness in Vietnam. Front. Vet. Sci..

[40] Schulz K., Conraths F.J., Blome S., Staubach C., Sauter-Louis C. (2019). African Swine Fever: Fast and Furious or Slow and Steady?. Viruses.

[41] de Carvalho Ferreira H.C., Backer J.A., Weesendorp E., Klinkenberg D., Stegeman J.A., Loeffen W.L.A. (2013). Transmission Rate of African Swine Fever Virus under Experimental Conditions. Vet. Microbiol..

[42] Guerra F.M., Bolotin S., Lim G., Heffernan J., Deeks S.L., Li Y., Crowcroft N.S. (2017). The Basic Reproduction Number (R0) of Measles: A Systematic Review. Lancet Infect. Dis..

[43] Costard S., Wieland B., de Glanville W., Jori F., Rowlands R., Vosloo W., Roger F., Pfeiffer D.U., Dixon L.K. (2009). African Swine Fever: How Can Global Spread Be Prevented?. Philos. Trans. R. Soc. B Biol. Sci..

[44] Villanueva-Cabezas J.P., Rajkhowa A., Campbell A.J.D. (2020). One Health Needs a Vision beyond Zoonoses. Transbound. Emerg. Dis..

[45] Cannon R.M. (2001). Sense and Sensitivity—Designing Surveys Based on an Imperfect Test. Prev. Vet. Med..

[46] Bradhurst R.A., Roche S.E., East I.J., Kwan P., Graeme Garner M. (2015). A Hybrid Modeling Approach to Simulating Foot-and-Mouth Disease Outbreaks in Australian Livestock. Front. Environ. Sci..

